# Importance of iron deficiency in patients with chronic heart failure as a predictor of mortality and hospitalizations: insights from an observational cohort study

**DOI:** 10.1186/s12872-018-0942-x

**Published:** 2018-11-01

**Authors:** José González-Costello, Josep Comín-Colet, Josep Lupón, Cristina Enjuanes, Marta de Antonio, Lara Fuentes, Pedro Moliner-Borja, Nuria Farré, Elisabet Zamora, Nicolás Manito, Ramón Pujol, Antoni Bayés-Genis

**Affiliations:** 1 Area de Enfermedades del Corazón, Hospital Universitari de Bellvitge, IDIBELL, Universitat de Barcelona, L’Hospitalet de Llobregat, Feixa Llarga SN, 08907 Barcelona, Spain; 2 Servicio de Cardiología, Hospital del Mar, IMIM, Universitat Autònoma de Barcelona, Barcelona, Spain; 3 Unidad de Insuficiencia Cardíaca, Hospital Universitari Germans Trias i Pujol, Universitat Autònoma de Barcelona, Badalona, Barcelona, Spain; 4 Servicio de Medicina Interna, Hospital Universitari de Bellvitge, IDIBELL, University of Barcelona, L’Hospitalet de Llobregat, Barcelona, Spain

**Keywords:** Chronic heart failure, Iron deficiency, Mortality, Hospitalization

## Abstract

**Background:**

Iron deficiency (ID) in patients with chronic heart failure (CHF) is considered an adverse prognostic factor. We aimed to evaluate if ID in patients with CHF is associated with increased mortality and hospitalizations.

**Methods:**

We evaluated ID in patients with CHF at 3 university hospitals. ID was defined as absolute (ferritin < 100 μg/L) or functional (transferrin Saturation index < 20% and ferritin between 100 and 299 μg/L). We excluded patients who received treatment with intravenous Iron or Erythropoietin during follow-up. We evaluated if ID was a predictor of death or hospitalization due to heart failure or any cause using univariate and multivariate cox regression analysis.

**Results:**

We included 1684 patients, 65% males, 38% diabetics, median age of 72 years, 37% in functional class III-IV and 30% of patients with a left ventricular ejection fraction > 45%. Patients were well treated, with 87% and 88% of patients receiving renin-angiotensin inhibitors and beta-blockers, respectively. Median transferrin saturation index was 20%, median ferritin 155 ng/mL and median haemoglobin 13 g/dL. ID was present in 53% of patients; in 35% it was absolute and in 18% functional. Median follow-up was 20 months. ID was a predictor of death, hospitalization due to heart failure or to any cause in univariate analysis but not after multivariate analysis. No differences were found between absolute or functional ID regarding prognosis.

**Conclusion:**

In a real life population of patients with CHF and a high prevalence of heart failure with preserved ejection fraction, ID did not predict mortality or hospitalizations after adjustment for comorbidities, functional class and neurohormonal treatment.

## Background

Iron deficiency (ID) affects up to 50% of patients with chronic heart failure (HF) and has been identified as an adverse prognostic factor independently of the presence of anaemia and chronic kidney disease (CKD) [1, 2]. However, in another study of community-dwelling adults with self-reported heart failure there was no association between ID and all cause or cardiovascular mortality, but haemoglobin and C-reactive protein did predict worse survival [3]. ID frequently overlaps with anaemia and/or CKD in chronic HF and the presence of ID amplifies mortality risk, either alone or in combination with anaemia, CKD, or both [2].

Although the pathophysiology behind ID in patients with chronic HF is not fully understood and is considered multifactorial [4–6], treatment of ID in these patients has become a therapeutic goal. Oral iron is not associated with an increase in exercise capacity in patients with HF and systolic dysfunction [7]. Recent randomized trials have demonstrated how intravenous iron can improve exercise capacity, cardiac function, symptom severity and quality of life in patients with chronic HF and left ventricular systolic dysfunction [8–11]. In the most recent study, the CONFIRM-HF, the risk of hospitalization due to worsening heart failure was reduced in the ferric carboxymaltose arm vs. the placebo-treated arm. However, no study has evaluated the incidence of hospitalizations due to heart failure or to any cause in patients with chronic HF and ID.

Given the importance of reducing mortality and hospitalizations in patients with chronic HF and that treatment with intravenous iron may reduce hospitalizations, we aimed to evaluate the prognostic importance of ID in a large multicenter cohort of ambulatory patients with chronic HF, focusing on mortality and hospitalizations due to worsening heart failure or to any cause.

## Methods

### Study design

This is a retrospective observational cohort study of patients with heart failure in whom we evaluated the presence of ID and whether ID was associated with re-hospitalizations or mortality during follow-up.

### Component population

The study population consisted of 2495 consecutive patients with stable chronic HF prospectively enrolled at 3 multidisciplinary heart failure (HF) units from 3 tertiary hospitals in Spain from 2005 to 2012. Patients had to be diagnosed of HF according to the European Society of Cardiology diagnostic criteria [12]. We defined HF with preserved LVEF (HFPEF) as the presence of symptoms of HF with a left ventricular ejection fraction ≥50% and an objective evidence of cardiac dysfunction (enlarged left atrium, diastolic dysfunction or elevated natriuretic peptides). We excluded patients without iron status assessed at entry and those with significant primary valvular or pericardial disease. Of the remaining 2172 patients, we excluded those who received treatment with intravenous iron or subcutaneous erythropoetin at any time point during follow-up, after review of the pharmacy records, as this treatment could influence outcomes. This left 1684 patients for analysis. Patients were treated according to the European Society of Cardiology guidelines [12].

Written informed consent was obtained from each patient. The investigation conforms with the principles outlined in the Declaration of Helsinki. The local ethic committees approved the study protocol.

### Pooled methodology

The pooled data for the present study were assessed at a patient level and collected prospectively. The end points for the present study were all cause mortality, first hospitalization due to HF and first hospitalization due to any cause after inclusion in the HF unit. Vital status and hospitalizations were assessed by review of the clinical databases, hospital records or direct contact with patients or relatives. Follow-up duration was until death or up to the last visit in the out patient clinic before October 2015. For patients lost to follow-up, we contacted the patient or the family by telephone and determined vital status and hospitalizations. For patients that could not be contacted by telephone we accessed the electronic clinical history of the hospital and of the primary care health system and determined vital status and hospitalizations. If despite all these efforts, no information regarding hospitalizations and death could be obtained, end of follow-up was determined to be the last time the patient had been to the out patient clinic. Five patients received a heart transplant during follow-up and this was recorded as death and end of follow-up. No patient received a ventricular assist device.

### Iron status and other laboratory measurements

The following blood biomarkers reflecting iron status were measured at study entry: Serum iron (ug/dL) was measured using spectrophotometry, serum ferritin (ng/mL) and transferrin (mg/dL) were measured using immunoturbidimetry. Transferrin saturation index (TSAT) was estimated using the formula: TSAT = serum iron (ug/dL)/[serum transferrin (mg/dL) × 1.25] [13]. ID was defined as absolute when ferritin < 100 ng/ml and functional when TSAT < 20% with ferritin 100–299 ng/ml [8].

Haemoglobin was measured using impedance laser colorimetry and anaemia was defined as Haemoglobin < 12 g/dL in women and Haemoglobin < 13 g/dL in men. Concentrations of N-terminal pro-brain-type natriuretic peptide (NT-proBNP) were measured using an immunoassay based on electrochemiluminescence on the Elecsys System (Roche Diagnostics, Basel, Switzerland). Renal function was assessed with the estimated glomerular filtration rate using the abbreviated Modification of Diet in Renal Disease equation.

### Statistical analysis

Continuous variables were explored for normal distribution according to histograms and the Shapiro-Wilk test. The comparison of quantitative variables between the 2 groups was done using the Student-t test for normally distributed variables and the Mann-Whitney U test for non-normally distributed variables. The chi-square test was used for categorical variables and Fisher’s exact test when cells had an expected count of less than 5.

The continuous variables that had a very skewed distribution, such as the TSAT, Ferritin and NT-proBNP underwent logarithmic transformation. The resulting variables are referred to as: logTSAT, logFerritin and logNT-proBNP. In order to assess if variables introduced in the multivariate regression analysis were highly correlated we evaluated collinearity using the variance inflation factor. The maximal variance inflation factor found among baseline variables was 1.5, indicating a low degree of collinearity.

To evaluate predictors of ID we used multivariate logistic regression analysis. All baseline variables with a significant univariate association (*p* < 0.10) or deemed to be clinically relevant were entered in a step-wise backward multivariable model with an exclusion criteria of *p* > 0.10. Additional bootstrap analysis (1000 cycles) of the multivariate model was performed to measure accuracy of the estimated model. The R2 value for the multivariate model was also calculated.

Incidence ratio of death and hospitalizations due to HF or any cause were determined dividing the incidence rate of events in patients with ID vs. without ID. Significance was assessed using the Mantel-Haenszel Chi square test.

Survival and time to first hospitalization due to heart failure or any cause were evaluated using Kaplan Meier survival curves. We evaluated if ID was a predictor of survival or hospitalization using univariate Cox proportional hazard regression analysis. With the variables that were significant in the univariate analysis (*p* < 0.10) and those that were deemed to be clinically relevant, we then performed backward step multivariate Cox proportional hazard analysis with an exclusion criteria of *p* > 0.10. ID is forced in to the final multivariate model given that it is the purpose of our study. The proportionality assumption for the Cox regression analysis was evaluated using residual analysis. Significance was set at *p* < 0.05 (2 tailed) and SPSS version 18 and Stata 14 were used to perform all statistical evaluations.

## Results

**Fig. 1:**
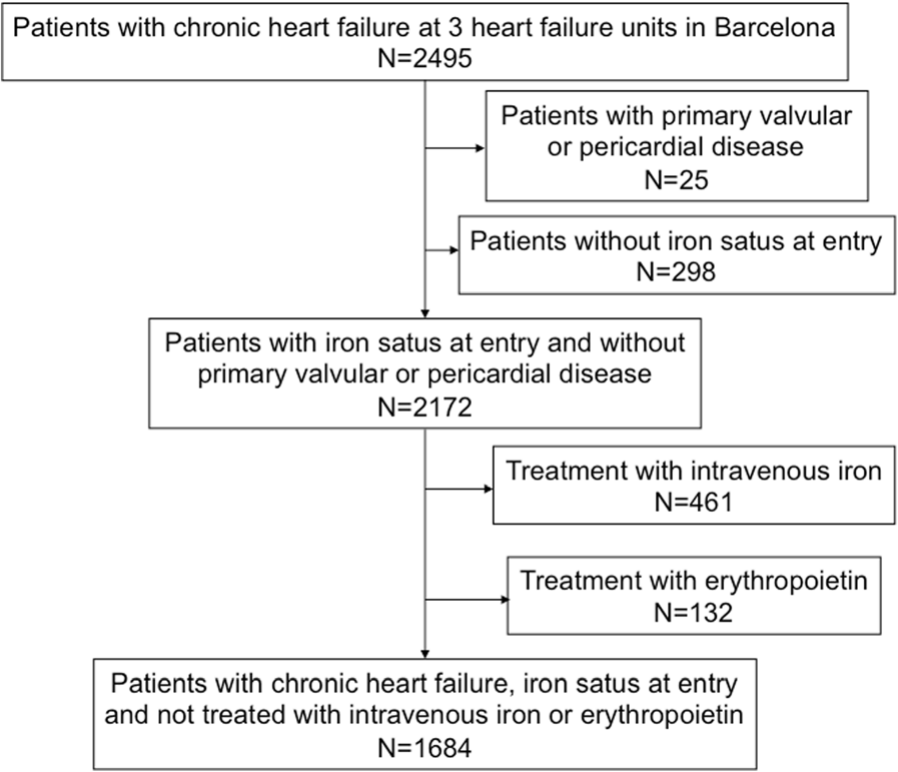
Flowchart of the study population. 105 of patients treated with erythropoietin were also treated with intravenous iron

We included 1684 patients in the analysis (see flowchart in Fig. 1), whose baseline characteristics are shown in Table 1. ID was present at inclusion in 898 (53%) patients. In 35% it was absolute ID and in 18% functional ID. There were 953 (57%) patients who were not anaemic and ID was present in 460 (48%) of these. Of the 731 patients with anaemia, ID was present in 438 (60%).

**Table 1:** Baseline variables according to iron deficiency

Variables	Overall *N* = 1684	No ID *N* = 786	ID *N* = 898	*P* value
Age (years)	72 (61–79)	70 (58–78)	73 (63–79)	< 0.0001
Female gender	588 (35)	207 (26)	381 (42)	< 0.0001
BMI (kg/m^2^)	27 (24–30)	30 (27–34)	31 (27–36)	0.011
Systolic BP (mm Hg)	120 (108–139)	118 (105–133)	125 (110–140)	< 0.0001
Heart rate (bpm)	70 (62–80)	70 (60–80)	70 (62–80)	0.398
NYHA Class III-IV	592 (35)	218 (28)	374 (42)	< 0.0001
LVEF (%)	35 (27–50)	35 (27–48)	36 (28–53)	0.032
HFPEF	428 (25)	182 (23)	246 (27)	0.046
Ischaemic aetiology	738 (44)	331 (42)	407 (45)	0.185
Hypertension	1136 (68)	492 (63)	644 (72)	< 0.0001
Diabetes Mellitus	639 (38)	262 (33)	377 (42)	< 0.0001
COPD	318 (19)	158 (20)	160 (18)	0.232
Treatment				
ACE-I or ARB	1457 (87)	695 (89)	762 (85)	0.019
Beta blockers	1474 (88)	690 (88)	784 (87)	0.766
MRAs	716 (43)	357 (45)	359 (40)	0.024
Digoxin	395 (23)	184 (23)	211 (24)	0.966
Oral anticoagulation	755 (45)	356 (45)	399 (44)	0.723
Antiplatelet	879 (52)	412 (52)	467 (52)	0.884
Diuretics	1458 (87)	661 (84)	797 (89)	0.001
ICD	136 (8)	64 (8)	72 (8)	0.933
Resynchronization	77 (5)	35 (5)	42 (5)	0.826
Laboratory values				
Haemoglobin (g/dL)	13.0 (11.7–14.3)	13.4 (12.0–14.7)	12.7 (11.5–13.9)	< 0.0001
eGFR (ml/min/1.73 m^2^)	53 (37–71)	56 (38–75)	52 (36–68)	0.032
Ferritin (ng/mL)	155 (72–276)	276 (172–435)	76 (43–141)	< 0.0001
Transferrin (mg/dL)	261 (224–313)	245 (213–288)	276 (239–330)	< 0.0001
Serum iron (ug/dL)	73 (51–99)	90 (71–116)	59 (44–79)	< 0.0001
TSAT (%)	20 (15–28)	26 (22–34)	16 (12–19)	< 0.0001
NTproBNP (ng/L)	1355 (536–3171)	1111 (488–2747)	1584 (609–3597)	< 0.0001
Sodium (mmol/L)	140 (138–142)	140 (138–142)	140 (138–142)	0.011

Continuous variables are expressed as median and interquartile range (IQR). Categorical variables are expressed as number and percentage

*ID* Iron deficiency, *BMI* Body mass index, *NYHA* New York Heart Association, *LVEF* Left ventricular ejection fraction, *HFPEF* Heart failure with preserved ejection fraction, *COPD* Chronic obstructive pulmonary disease, *ACE-I* Angiotensin converting enzyme inhibitor, *ARB* Angiotensin II receptor blocker, *MRA* Mineralocorticoid receptor antagonist, *ICD* Implantable cardioverter defibrillator, *eGFR* estimated glomerular filtration rate, *TSAT* Transferrin saturation, *NTproBNP* N-terminal pro-brain natriuretic peptide

Predictors of ID after multivariate analysis are shown in Table 2. The R2 for the multivariate model was 0.05 (5%). Median follow-up was 20 (interquartile range: 12–47) months.

**Table 2:** Predictors of iron deficiency using logistic regression analysis

Variables	Univariate OR	*P* value	Multivariate OR	*P* value
Age (per 5 years)	1.09 (1.05–1.13)	< 0.001		
Female sex	2.05 (1.67–2.53)	0.396	1.62 (1.29–2.03)	< 0.001
Hypertension	1.50 (1.23–1.85)	< 0.001		
Diabetes Mellitus	1.44 (1.18–1.76)	< 0.001		
COPD	0.86 (0.67–1.10)	0.223		
HFPEF	1.25 (1.00–1.56)	0.047		
Ischaemic aetiology	1.15 (0.94–1.39)	0.175		
NYHA class III-IV	1.84 (1.50–2.26)	< 0.001	1.50 (1.20–1.88)	< 0.001
Systolic BP (per 10 mmHg)	1.11 (1.06–1.16)	< 0.001	1.09 (1.04–1.14)	< 0.001
Heart rate (per 10 bpm)	1.03 (0.97–1.10)	0.379		
BMI (1 kg/m^2^)	1.03 (1.01–1.04)	0.008	1.03 (1.00–1.05)	0.018
ACE-I or ARB	0.71 (0.53–0.95)	0.021		
Beta blockers	0.96 (0.72–1.28)	0.781		
Diuretics	1.50 (1.13–1.98)	0.005		
MRAs	0.81 (0.66–0.98)	0.028		
Digoxin	1.00 (0.80–1.26)	0.988		
Oral anticoagulants	0.96 (0.79–1.17)	0.690		
Antiplatelet	0.99 (0.82–1.20)	0.921		
ICD	1.02 (0.71–1.44)	0.94		
Haemoglobin (per 1 g/dL)	0.82 (0.78–0.87)	< 0.001	0.87 (0.82–0.92)	< 0.001
Log NT-proBNP (per 1 SD)	1.20 (1.09–1.33)	< 0.001	1.12 (0.99–1.25)	0.051
Sodium (per 5 mmol/L)	1.17 (1.02–1.34)	0.030		
eGFR (per 5 ml/min/1.73m^2^)	0.98 (0.96–0.99)	0.016		

*OR* Odds Ratio, *COPD* Chronic Obstructive Pulmonary Disease, *HFPEF* Heart failure with preserved ejection fraction, *NYHA* New York Heart Association, *BP* Blood pressure, *BMI* Body mass index, *ACE-I* Angiotensin converting enzyme inhibitor, *ARB* Angiotensin II receptor blocker, *MRAs* Mineralocorticoid receptor antagonists, *ICD* Implantable cardioverter defibrillator, *Log* Logarithmic transformation, *NTproBNP* N-terminal pro-brain natriuretic peptide, *SD* Standard deviation, *eGFR* Estimated glomerular filtration rate

Variables introduced in the multivariate model were: Age, gender, hypertension, diabetes mellitus, COPD, HFPEF, NYHA functional class III-IV, systolic BP, heart rate, BMI, haemoglobin, logNT-proBNP, eGFR, sodium, treatment with beta blockers, ACE-i/ARBs, diuretics and MRAs

### Iron deficiency and mortality

**Fig. 2:**
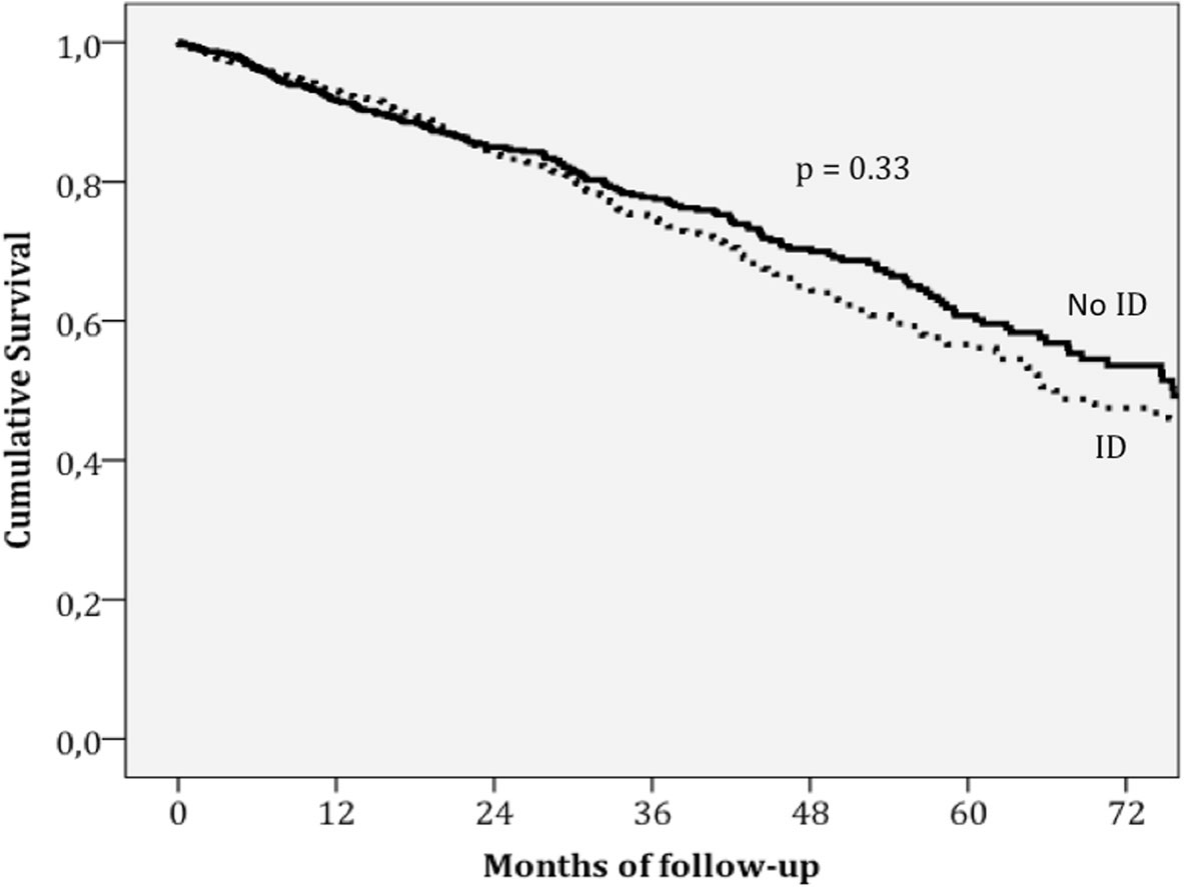
Survival curves stratified for ID after adjustment for the covariates that are significant in the multivariate model: Age, sex, diabetes mellitus, New York Heart Association class III-IV, haemoglobin, logarithmic transformation of N-terminal pro-brain natriuretic peptide, serum sodium, and treatment with an angiotensin converting enzyme inhibitor or angiotensin II receptor blocker, beta blockers and diuretics

Patients with ID had 307 (34%) deaths with an incidence rate of 0.134 per year compared with those without ID, who had 212 (27%) deaths, with an Incidence Rate of 0.101 per year. The incidence ratio was 1.32 (95% confidence interval: 1.11–1.57; *p* = 0.0017). Both absolute and functional ID had similar mortality rates of 0.145 and 0.128 per year, respectively, *p* = 0.29. When we analysed both components of the definition of ID, lower logTSAT predicted mortality but not logFerritin. ID was not a predictor of mortality in multivariate analysis. Table 3 shows the hazard ratios for mortality in our cohort of patients using univariate and multivariate cox regression analysis. When we introduced logTSAT in the multivariate model, instead of the usual definition of ID, it also was not a significant predictor of mortality. Figure 2 shows no differences in the survival curves stratified for ID after adjustment for the covariates that were significant in the multivariate model.

**Table 3:** Predictors of mortality using cox regression analysis

Variables	Univariate HR	*P* value	Multivariate HR	*P* value
Age (per 5 years)	1.36 (1.30–1.42)	< 0.001	1.21 (1.16–1.27)	< 0.001
Female sex	1.08 (0.90–1.30)	0.39	0.65 (0.54–0.79)	< 0.001
Hypertension	1.35 (1.12–1.63)	0.002		
Diabetes Mellitus	1.51 (1.27–1.80)	< 0.001	1.30 (1.08–1.55)	0.005
COPD	1.63 (1.33–1.99)	< 0.001		
HFPEF	1.11 (0.90–1.36)	0.34		
Ischaemic aetiology	1.09 (0.92–1.30)	0.33		
NYHA class III-IV	3.14 (2.63–3.74)	< 0.001	1.85 (1.53–2.24)	< 0.001
Systolic BP (per 10 mmHg)	0.97 (0.93–1.01)	0.112		
Heart rate (per 10 bpm)	1.09 (1.03–1.16)	0.004		
BMI (1 kg/m^2^)	0.96 (0.95–0.98)	< 0.001		
ACE-I or ARB	0.37 (0.29–0.46)	< 0.001	0.70 (0.55–0.89)	0.004
Beta blockers	0.41 (0.33–0.51)	< 0.001	0.59 (0.47–0.74)	< 0.001
Diuretics	2.46 (1.77–3.42)	< 0.001	1.66 (1.19–2.31)	0.005
MRAs	0.97 (0.82–1.16)	0.77		
Digoxin	1.26 (1.05–1.52)	0.015		
ICD	0.72 (0.52–0.98)	0.039		
Iron deficiency	1.32 (1.11–1.57)	0.002	1.09 (0.91–1.31)	0.337
Haemoglobin (per 1 g/dL)	0.78 (0.75–0.82)	< 0.001	0.90 (0.85–0.95)	< 0.001
Log NT-proBNP (per 1 SD)	1.93 (1.76–2.11)	< 0.001	1.49 (1.34–1.66)	< 0.001
Sodium (per 5 mmol/L)	0.78 (0.69–0.88)	< 0.001	0.96 (0.94–0.99)	0.003
eGFR (per 5 ml/min/1.73m^2^)	0.89 (0.87–0.91)	< 0.001		

*HR* Hazard Ratio, *COPD* Chronic Obstructive Pulmonary Disease, *HFPEF* Heart failure with preserved ejection fraction, *NYHA* New York Heart Association, *BP* Blood pressure, *BMI* Body mass index, *ACE-I* Angiotensin converting enzyme inhibitor, *ARB* Angiotensin II receptor blocker, *MRAs* Mineralocorticoid receptor antagonists, *ICD* Implantable cardioverter defibrillator, *Log* Logarithmic transformation, *NTproBNP* N-terminal pro-brain natriuretic peptide, *SD* Standard deviation, *eGFR* Estimated glomerular filtration rate

Variables introduced in the multivariate model were: Age, gender, ischaemic aetiology, systolic BP, HFPEF, diabetes mellitus, hypertension, COPD, BMI, NYHA functional class III-IV, haemoglobin, logNT-proBNP, eGFR, sodium, heart rate, treatment with beta blockers, ACE-i/ARBs, diuretics, digoxin and an ICD

### Iron deficiency and hospitalizations due to heart failure

**Fig. 3:**
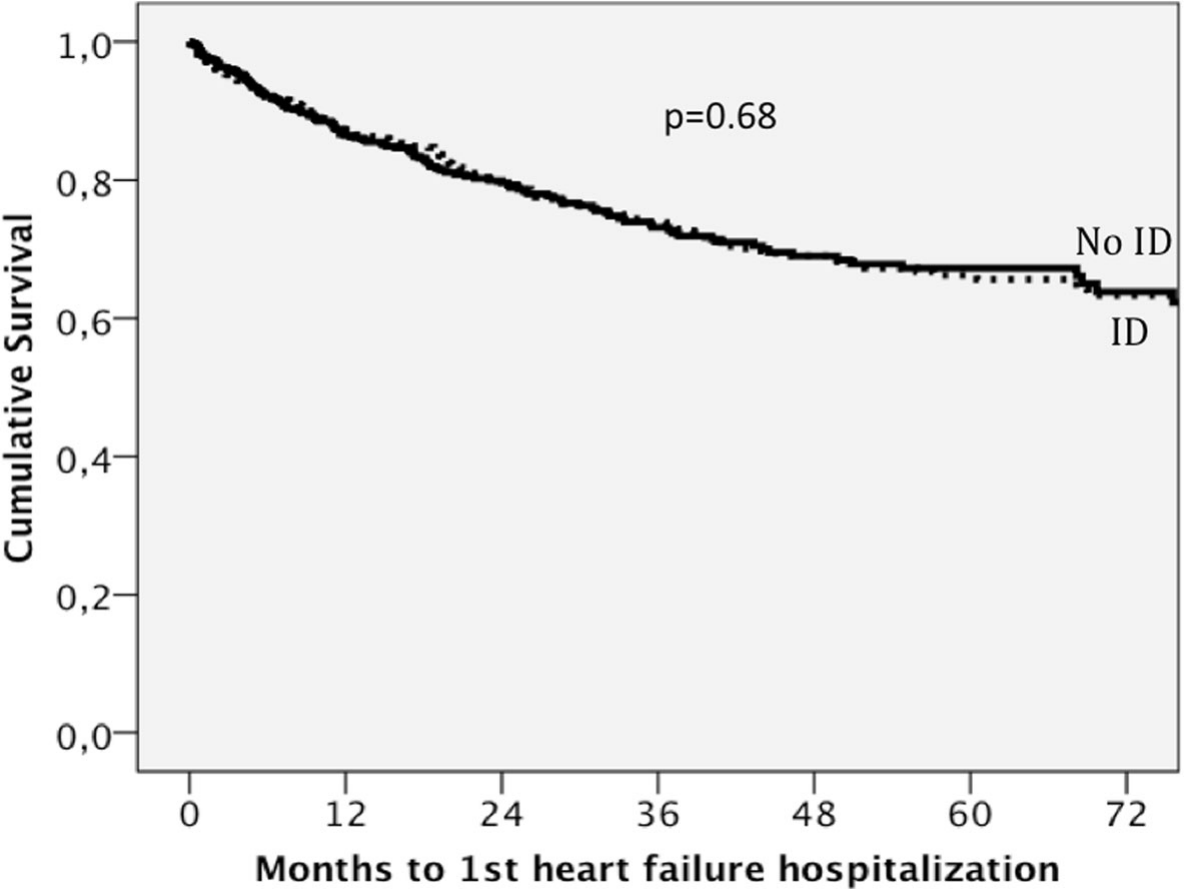
Survival curves for hospitalization due to HF stratified for ID after adjustment for the covariates that are significant in the multivariate model: Age, diabetes mellitus, chronic pulmonary obstructive disease, heart failure with preserved ejection fraction, ischaemic aetiology, New York Heart Association class III-IV, logarithmic transformation of N-terminal pro-brain natriuretic peptide, and treatment with an angiotensin converting enzyme inhibitor or angiotensin II receptor blocker, beta blockers and diuretics

Patients with ID had 246 (27%) hospitalizations due to HF with an incidence rate of 0.121 per year compared with those without ID, who had 182 (23%) hospitalizations due HF, with an incidence rate of 0.097 per year. The incidence ratio was 1.24 (95% confidence interval: 1.03–1.51; *p* = 0.025). There was no significant difference in the incidence rate of hospitalizations due to heart failure between patients with functional or absolute ID: 0.110 and 0.125 per year respectively, *p* = 0.37. ID was not a predictor of hospitalization due to heart failure after multivariate analysis. Table 4 shows predictors of hospitalization due to HF after univariate and multivariate adjustment. Figure 3 shows the survival curves for hospitalization due to HF stratified for ID and adjusted for the covariates that were significant in the multivariate model.

**Table 4:** Predictors of heart failure hospitalization using Cox regression analysis

Variables	Univariate HR	*P* value	Multivariate HR	*P* value
Age (per 5 years)	1.23 (1.18–1.29)	< 0.001	1.11 (1.05–1.17)	< 0.001
Female sex	1.50 (1.25–1.83)	< 0.001		
Hypertension	1.47 (1.19–1.81)	< 0.001		
Diabetes Mellitus	1.46 (1.20–1.77)	< 0.001	1.26 (1.03–1.54)	0.026
COPD	1.76 (1.41–2.19)	< 0.001	1.63 (1.28–2.07)	< 0.001
HFPEF	1.60 (1.30–1.97)	< 0.001	1.67 (1.31–2.18)	< 0.001
Ischaemic aetiology	1.14 (0.95–1.38)	0.166	1.40 (1.12–1.73)	0.003
NYHA class III-IV	2.55 (2.10–3.09)	< 0.001	1.64 (1.33–2.03)	< 0.001
Systolic BP (per 10 mmHg)	0.97 (0.93–1.01)	0.123		
Heart rate (per 10 bpm)	1.09 (1.03–1.16)	0.005		
BMI (per 1 kg/m^2^)	0.99 (0.97–1.01)	0.21		
ACE-I or ARB	0.47 (0.37–0.61)	< 0.001		
Beta blockers	0.46 (0.36–0.58)	< 0.001	0.71 (0.54–0.93)	0.013
Diuretics	3.47 (2.28–5.28)	< 0.001	2.53 (1.64–3.91)	< 0.001
MRAs	1.10 (0.91–1.33)	0.327		
Digoxin	1.13 (0.91–1.40)	0.263		
ICD	0.91 (0.66–1.27)	0.581		
Iron deficiency	1.21 (1.00–1.47)	0.047	0.96 (0.78–1.17)	0.677
Haemoglobin (per 1 g/dL)	0.84 (0.80–0.89)	< 0.001		
Log NT-proBNP (per 1 SD)	2.00 (1.87–2.15)	< 0.001	1.49 (1.32–1.68)	< 0.001
Sodium (per 5 mmol/L)	0.93 (0.81–1.07)	0.328		
eGFR (per 5 ml/min/1.73m^2^)	0.95 (0.93–0.97)	< 0.001		

*HR* Hazard Ratio, *COPD* Chronic Obstructive Pulmonary Disease, *HFPEF* Heart failure with preserved ejection fraction, *NYHA* New York Heart Association, *BP* Blood pressure, *BMI* Body mass index, *ACE-I* Angiotensin converting enzyme inhibitor, *ARB* Angiotensin II receptor blocker, *MRAs* Mineralocorticoid receptor antagonists, *ICD* Implantable cardioverter defibrillator, *Log* Logarithmic transformation, *NTproBNP* N-terminal pro-brain natriuretic peptide, *SD* Standard deviation, *eGFR* Estimated glomerular filtration rate

Variables introduced in the multivariate model were gender, age, diabetes mellitus, hypertension, BMI, ischaemic aeetiology, systolic blood pressure, COPD, HFPEF, heart rate, NYHA functional class III-IV, haemoglobin, logNT-proBNP, eGFR and treatment with ACE-i/ARBs, beta blockers, diuretics and MRAs

### Iron deficiency and hospitalizations due to any cause

Patients with ID had 465 (52%) hospitalizations due to any cause with an Incidence Rate of 0.284 per year compared with those without ID, who had 363 (46%) hospitalizations due to any cause, with an incidence rate of 0.226 per year. The incidence ratio was 1.26 (95% confidence interval: 1.09–1.44; *p* = 0.001).

**Fig. 4:**
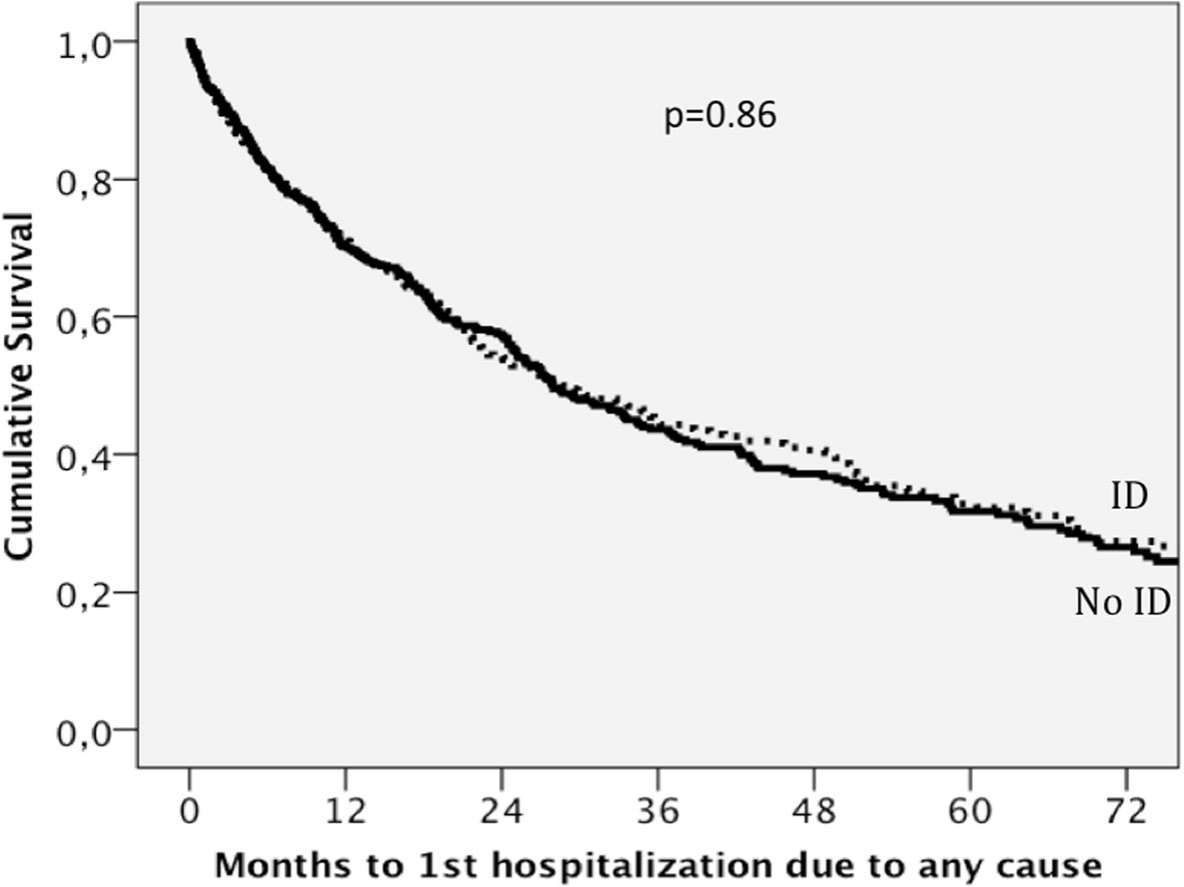
Survival curves for hospitalization due to any cause stratified for ID after adjustment for the covariates that are significant in the multivariate model: Age, diabetes mellitus, chronic pulmonary obstructive disease, heart failure with preserved ejection fraction, New York Heart Association class III-IV, heart rate, haemoglobin, logarithmic transformation of N-terminal pro-brain natriuretic peptide, and treatment with an angiotensin converting enzyme inhibitor or angiotensin II receptor blocker, beta blockers and diuretics

Patients with functional ID had a rate of hospitalizations due to any cause of 0.266 per year compared with 0.290 per year in those with absolute ID, *p* = 0.36. ID was not a predictor of hospitalization due to any cause after multivariate analysis. Table 5 shows the predictors of hospitalization due to any cause after univariate and multivariate adjustment. Figure 4 shows the survival curves for hospitalization due to any cause stratified for ID and adjusted for the covariates that were significant in the multivariate model.

**Table 5:** Predictors of hospitalization due to any cause using Cox regression analysis

Variables	Univariate HR	*P* value	Multivariate HR	*P* value
Age (per 5 years)	1.17 (1.13–1.20)	< 0.001	1.06 (1.02–1.09)	0.001
Female sex	1.29 (1.12–1.45)	< 0.001		
Hypertension	1.42 (1.22–1.65)	< 0.001		
Diabetes Mellitus	1.46 (1.27–1.68)	< 0.001	1.23 (1.06–1.42)	0.006
COPD	1.56 (1.32–1.84)	< 0.001	1.44 (1.21–1.71)	< 0.001
HFPEF	1.40 (1.20–1.63)	< 0.001	1.44 (1.21–1.73)	< 0.001
Ischaemic aetiology	1.16 (1.01–1.33)	0.032	1.32 (1.14–1.54)	< 0.001
NYHA class III-IV	2.03 (1.77–2.34)	< 0.001	1.40 (1.20–1.64)	< 0.001
Systolic BP (per 10 mmHg)	1.01 (0.98–1.04)	0.522		
Heart rate (per 10 bpm)	1.13 (1.08–1.19)	< 0.001	1.07 (1.02–1.12)	0.006
BMI (per 1 kg/m^2^)	0.99 (0.97–0.99)	0.033		
ACE-I or ARB	0.50 (0.41–0.61)	< 0.001	0.80 (0.65–0.98)	0.030
Beta blockers	0.51 (0.43–0.62)	< 0.001	0.69 (0.57–0.86)	0.001
Diuretics	1.70 (1.36–2.12)	< 0.001	1.31 (1.04–1.65)	0.022
MRAs	0.95 (0.83–1.09)	0.488		
Digoxin	1.01 (0.86–1.18)	0.940		
ICD	0.88 (0.69–1.12)	0.284		
Iron deficiency	1.21 (1.05–1.39)	0.007	0.99 (0.86–1.14)	0.857
Haemoglobin (per 1 g/dL)	0.85 (0.81–0.88)	< 0.001	0.94 (0.90–0.98)	0.002
LogNT-proBNP (per 1 SD)	1.53 (1.42–1.64)	< 0.001	1.31 (1.20–1.42)	< 0.001
Sodium (per 5 mmol/L)	0.89 (0.80–0.98)	0.023		
eGFR (per 5 ml/min/1.73m^2^)	0.95 (0.94–0.96)	< 0.001		

*HR* Hazard Ratio, *COPD* Chronic Obstructive Pulmonary Disease, *HFPEF* Heart failure with preserved ejection fraction, *NYHA* New York Heart Association, *BP* Blood pressure, *bpm* beats per minute, *BMI* Body mass index, *ACE-I* Angiotensin converting enzyme inhibitor, *ARB* Angiotensin II receptor blocker, *MRAs* Mineralocorticoid receptor antagonists, *ICD* Implantable cardioverter defibrillator, *Log* Logarithmic transformation, *NTproBNP* N-terminal pro-brain natriuretic peptide, *SD* Standard deviation, *eGFR* Estimated glomerular filtration rate

We included the following variables in the multivariate analysis: Haemoglobin, sex, age, HFPEF, BMI, Diabetes Mellitus, Hypertension, COPD, ischaemic aetiology, logNTproBNP, eGFR, serum sodium, systolic BP, heart rate, NYHA functional class III-IV and treatment with ACE-I/ARB, Beta-blockers and diuretics

## Discussion

The main findings of our study are:

1. ID was present in more than half of a large cohort of ambulatory patients with chronic HF.
2. ID did not predict mortality after multivariate adjustment.
3. ID did not predict increased hospitalizations due to heart failure or due to any cause after multivariate adjustment.

ID was very prevalent in our cohort of patients with chronic HF and most of it was due to absolute ID. Predictors of ID after multivariate analysis were female gender, a higher body mass index, more advanced functional class, increased systolic blood pressure and lower haemoglobin values. These findings are comparable to previous findings from other groups [1–3, 14].

### Iron deficiency and mortality

ID has been shown to be a marker of increased mortality independently of anaemia and CKD [1, 2, 14], but in our study we were not able to demonstrate it after multivariate analysis. However haemoglobin remained a significant predictor of mortality, as in the study by Parikh et al. [3]. We can only hypothesize why this is, but one major difference is that in our study the number of patients with HFPEF was significant compared with none in the study by Jankowska et al. [1] and 13% in the study by Klip et al. [14]. In the study by Parikh et al. [3] this data is unknown but given the relatively high mean systolic blood pressure, large proportion of women and age of the population it is likely that many of the participants had HFPEF. When we analyzed if ID was a predictor of mortality only in patients with systolic dysfunction, in our cohort it was not significant in multivariate analysis. Our patients were more likely to be older and had more CKD and anaemia compared to those studied in the 3 previously mentioned cohorts and this may imply that other factors besides ID may be more important in predicting mortality in our patients. The variables that predicted mortality in our patients are otherwise not different from those previously described in the literature [9, 15].

We used the definition of ID according to the FAIR-HF trial [8]. However, diagnosis based on TSAT and ferritin may be inadequate in advanced disease or acute HF, where ferritin becomes an unreliable marker [16]. In our cohort of ambulatory patients with chronic HF, ferritin did not predict mortality in univariate analysis and the driver of adverse prognosis was the TSAT.

### Iron deficiency and hospitalizations

Hospitalizations due to HF are frequent in patients with chronic HF and linked with increased mortality [17], impairment of patient’s quality of life and an economic burden for the health system [18]. Although ID has been shown to be a key determinant of health-related quality of life in patients with chronic HF [19], we are not aware that the association between ID and increased risk of hospitalizations due to HF or due to any cause has been studied in patients with chronic HF. Our study is the first to do so, and we have shown that ID was associated with hospitalizations due to HF in univariate analysis but not after multivariate analysis. When we looked at predictors of hospitalization due to HF we found that they were very similar to those that predicted mortality. The only difference being that lower serum sodium and lower haemoglobin predicted mortality but not hospitalizations due to HF. On the other hand, chronic obstructive pulmonary disease, HFPEF and ischaemic etiology were associated with increased risk of hospitalization due to HF but not with mortality. Patients with chronic HF such as those seen in our study and in recent registries have many comorbidities [20], and hospitalizations may be related to these comorbidities. Therefore, we thought it would be interesting to look at hospitalizations due to any cause. ID was a predictor of hospitalizations due to any cause in univariate analysis, but again not after multivariate analysis. Predictors of hospitalization due to any cause were multiple and included all that predicted mortality and all that predicted hospitalizations due to HF, except for female sex and serum sodium, but with the addition of heart rate. What stands out from our analysis is that HFPEF was associated with a higher risk of hospitalizations due to heart failure or to any cause, and this may be related not so much to heart failure itself but to the increased cardiovascular and non-cardiovascular comorbidities of patients with HFPEF [21]. One comorbidity that is particularly prevalent in HFPEF is chronic pulmonary obstructive disease [22], and it is notable that in our cohort it was strongly associated with increased hospitalizations due to HF and to any cause but not with mortality, as has been shown in previous studies of patients with HF [23, 24].

It is plausible that in our cohort, other factors might diminish the importance of ID as a predictor of hospitalizations. However, our patient population clearly resembles the usual patients seen in clinical practice [18] and our results issue a word of caution in extrapolating that ID is linked with increased hospitalizations either due to HF or to any cause. Therefore, further studies are needed to evaluate this association, and in this direction, a report linking ID with 30 days readmission in patients with acute heart failure was recently published [25].

### Iron deficiency in non-anaemic patients

In the FAIR-HF trial, treatment of ID with ferric carboximaltose in patients with chronic HF was equally efficacious in anaemic and in non-anaemic patients [26]. Given the fact that in our study, anaemia but not ID predicted mortality, we explored the association of ID with prognosis in non-anaemic patients. Although ID predicted increased mortality and hospitalizations in univariate analysis, this association did not hold up in multivariate analysis.

### Limitations

We did not measure other markers of ID, such as the soluble transferrin receptor, hepcidin or the ferritin index that may better evaluate iron metabolism in patients with chronic heart failure [5, 27, 28]. We also did not assess C-reactive protein, high-sensitivity cardiac troponin T, and high-sensitivity soluble ST2, which have been associated with increased mortality in patients with chronic HF [3, 15]. We had no information regarding other non-cardiovascular comorbidities such as cancer or liver disease that could be potential confounders of outcomes in such an elderly population.

We excluded from the analysis patients that received treatment with intravenous iron or erythropoietin, creating a possible bias. However, this treatment was not approved by the guidelines at the time the study was performed and given that both these treatments could influence ID, we preferred to exclude these patients.

When assessing hospitalizations we did not look at recurrent number of hospitalizations, and this is also an important outcome that influences quality of life in patients with HF.

## Conclusions

In a contemporary cohort of patients with chronic HF, ID was present in more than half of the patients. However, its presence was not a predictor of mortality or hospitalizations due to HF or to any cause after multivariate adjustment. Further studies are needed to assess the prognostic significance of ID in real life patients with chronic HF and where HFPEF is increasingly prevalent.

## Abbreviations

CKD: Chronic kidney disease
HF: Heart failure
HFPEF: Heart failure with preserved ejection fraction
ID: Iron deficiency
NT-proBNP: N-terminal pro-brain-type natriuretic peptide
TSAT: Transferrin saturation index
